# Supplementary material for: Control of nuclear dynamics in the benzene cation by electronic wavepacket composition
Source: Commun Chem. 2021 Apr 1;4:48. doi: 10.1038/s42004-021-00485-3 (PMC9814899; doi:10.1038/s42004-021-00485-3)
Supplement: Supplementary file 1 — Supplementary Information [file 42004_2021_485_MOESM1_ESM.pdf]

Control of Nuclear Dynamics in the Benzene Cation by Electronic Wavepacket Composition

Thierry Tran[1,2 a)], Graham A. Worth[1,b)], and Michael A Robb[2,c)]

[1] *Department of Chemistry, University College London, 20, Gordon St., WC1H 0AJ London, United-Kingdom*

[2] *Department of Chemistry, Molecular Sciences Research Hub, Imperial College London, White City Campus, 80 Wood Lane, W12 0BZ, London, United-Kingdom*

Supplementary Note 1

The Qu-Eh method solves the time-dependent Schrödringer equation for both CI coefficients and nuclear wavefunction expansion coefficients. The equations for the time-evolution are variational[1] but the orbitals are only propagated to second order. The electronic wavefunction (see equation 1 in main text) is combined with the nuclear wavefunction which is expanded in gaussian wavepackets (see equation S1 below)

$$g_i(R,t) = \exp\left(-\alpha(t)\big(R - R_i(t)\big)^2 + iP_i(t)\big(R - R_i(t)\big) + \gamma_i(t)\right) \qquad \text{(S1)}$$

where R is the nuclear coordinate and α, R_i, P_i and γ_i are respectively, the width, position of centre, momentum of centre and phase of gaussian wavepacket i. In the derivation in ref. 1, the time-dependent parameters of the gaussian wavepackets are collected together in the vectors Λ_i.

By inserting the ansatz into the Dirac-Frenkel variational principle,

$$< \delta\Psi|H - i\frac{\partial}{\partial t}|\Psi> = 0 \qquad \text{(S2)}$$

we obtain equation of motions for the expansion coefficients A,

$$i\dot{A}_k = \sum_{ij} S_{ki}^{-1}\big(H_{ij}^N - i\tau_{ij}\big)A_j \qquad \text{(S3)}$$

the gaussian wavepacket parameters Λ,

$$i\dot{\Lambda}_{\beta k} = \sum_{\alpha ij} C_{\beta k\alpha i}^{-1}\,\rho_{ij}\left(H_{ij}^{N(\alpha 0)} - \sum_{lm} S_{il}^{(\alpha 0)} S_{lm}^{-1} H_{mj}^N\right) \qquad \text{(S4)}$$

and the electronic state coefficient c

$$i\dot{c}_s = \sum_t \big(H_{el,st} - \delta_{st}V\big)c_t. \qquad \text{(S5)}$$

The parameters are defined as follows: α and β are the indices of the nth parameter of gaussian wavepacket and i, j and k are the indicies of the gaussian wavepacket. S is the overlap matrix between gaussian wavepackets, H^N the nuclear Hamiltonian, τ the time-derivative overlap matrix, C the DD-vMCG C matrix (see ref. 2 main paper), H_{el} the electronic hamiltonian and V the Ehrenfest potential. The exponent $\alpha 0$ means derivative of the gaussian function on the left side with respect to the αth parameter.

In Qu-Eh, the nuclear Hamiltonian is evaluated using a local harmonic expansion around the centre of the individual gaussian wavepacket. The 2nd order expansion is done by evaluating the energy, gradient and Hessian at each gaussian centre for each nuclear time step. The main steps in the algorithm for Qu-Eh for each time step are:

1. The geometry for the centre of each gwp in cartesian coordinates corresponds to the usual electronic structure input
2. Integrate equations of motion for electronic state coefficients with electronic structure method (electron dynamics):
   2.1. Propagate the configuration interaction coefficients with CAS-CI
   2.2. Evaluate the Ehrenfest energy
   2.3. Evaluate the Ehrenfest gradient and Hessian (including all off diagonal terms and solving the coupled perturbed equations for orbial and CI parameters
3. Build nuclear Hamiltonian in the gaussian wavepacket basis using a local harmonic approximation
4. Integrate the equation of motions for gaussian parameters and expansion coefficients
5. Repeat from 1

Supplementary Note 2

The initial "stationary" nuclear wavepacket is built by using a Gaussian function centred at the intial geometry using a local harmonic approximation (from the 1st and 2nd derivatives obtained from a frequency calculation at that geometry) of the neutral ground state S_0 potential energy surface.

The 25 gwp are distributed in phase space with a momentum distribution where the gwp form a shell around the centre position. The gwp 1 has no initial momentum and the gwp 2 to 12 are placed in phase space by initially exciting

one degree of freedom in a sequential manner, *i.e.* they have an initial momentum put in one specific normal mode (+ and – to give 25 gwp). The width of the gwp is 0.1 (in dimensionless unit).

In our computations, the expectation value of the momentum for each normal mode, <p> is zero. However, each individual gwp has a momentum and each gwp is initially associated with a normal mode. There are 2 gwp associated with related each vibrational coordinate, one with +p and one –p where p =11.77. Note that p is related to the dimensionless mass-frequency scaled normal co-ordinates and has units of inverse time. The value is chosen so that the overlap with the central gwp is 0.8. The value of p for each gwp is identical and the initial momentum for gwp 1 is 0.

The initial weight of each gwp is defined by fitting the initial "stationary" nuclear wavepacket as accurately as possible. By using a smaller width (.1 rather than .707) for the gwp, the initial nuclear wavepacket is also narrowed.

Supplementary Note 3

Symmetries of the MO of the 8 cationic states

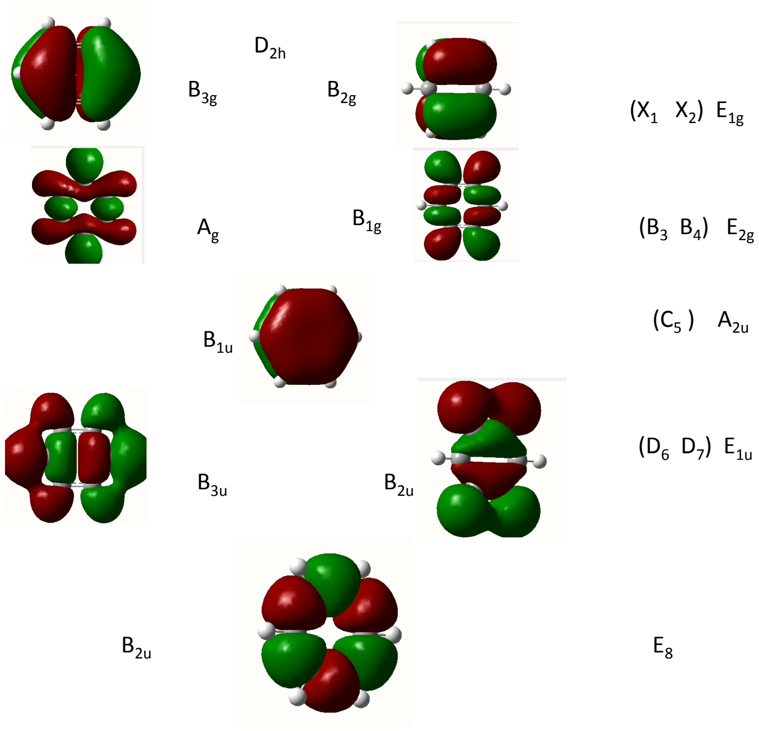

Figure S1 Symmetries of the MO of the 8 cationic states of benzene cation : Shown are the MO from which the electron is removed and the corresponding symmetries in D_{6h} and D_{2h} (we choose the principal axis in both groups to be the z-axis). The numbers 1-8 correspond to energy ordering. (Except that the pairs of states D_6 and $D_{7,}$ B_3 B_4 and X_2 X_1, have the same energy and belong to the degenerate E_{1u} or E_{1g} representations)

Supplementary Note 4

Normal mode symmetries and direct products

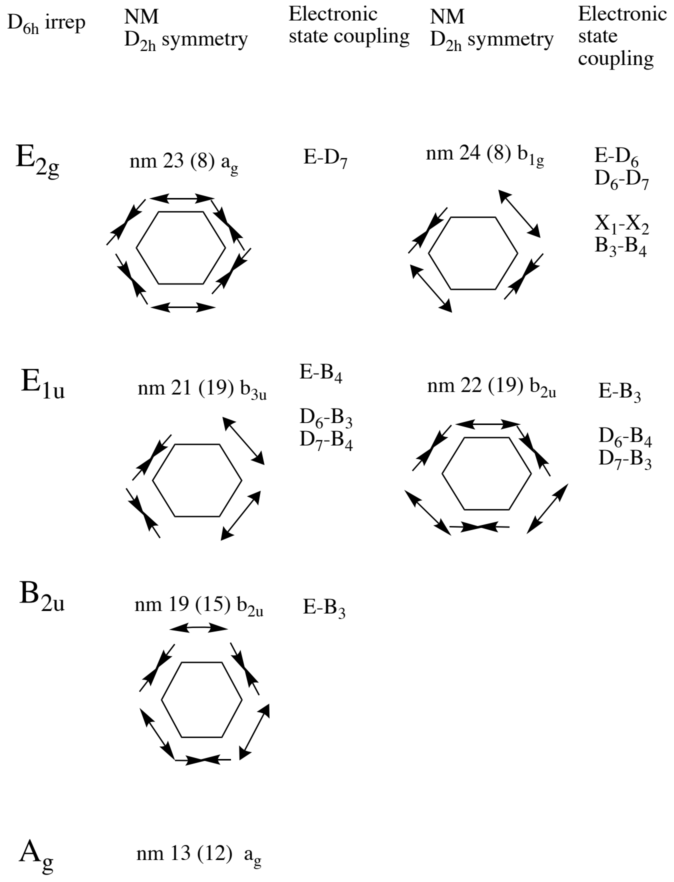

Figure S2 Normal mode symmetries and direct products of electronic states that yield these symmetries. The nm are ordered by energy (from ground state B3LYP frequency computation). The numbers in brackets correspond the modes used in supplementart reference 2.

Supplementary Note 5

Electron dynamics E B_3

In figure   S3 we illustrate the spin density oscillations E + B_3 on the 6 C atoms together with a schematic illustration of the electron density (parts a b and c). Note the node through atoms 1 and 4 . Once can observe that the spin density on atoms 3 and 5 are out of phase

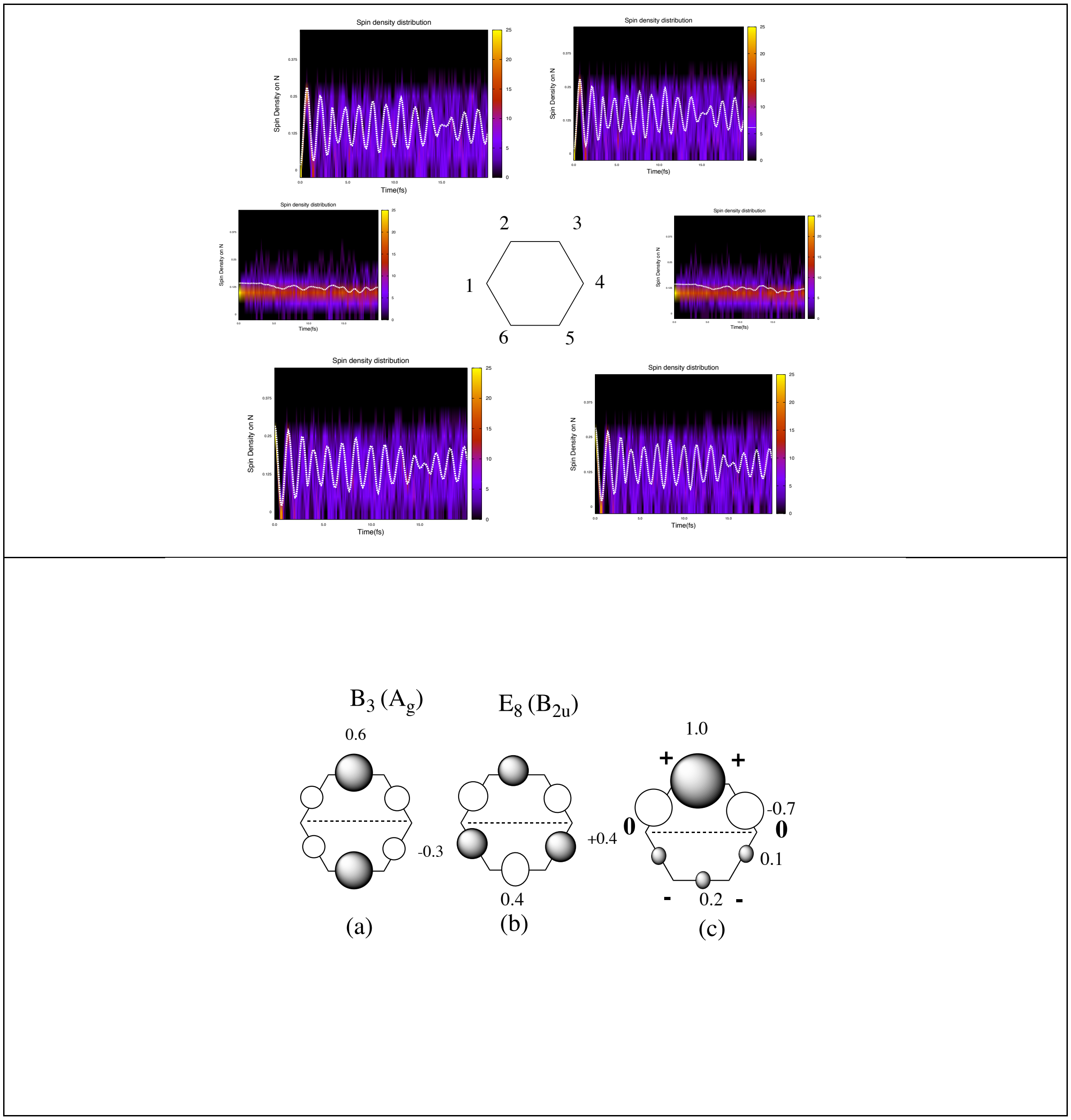

Figure S3.   Electron Dynamics. The spin density (top) as a function of time, centred on 10fs. stimulated by E + B_3.   Each plot gives the spin density on atoms 1 thru 6. The schematic bond (bottom) electron density of E and B_3 is shown in parts a and b with the sum in part c. The dashed line shows the node passing through atoms 1 and 4 (zero spin density). The solid/open circle on a b and c indicates a

positive/negative electron density. The period of oscillation is approximately 0.25 fs. Notice the spin density on atoms 2 and 3 is in phase and out of phase between atoms 2 and 6 or 3 and 5 in agreement with part c.

Supplementary References

1)Theor Chem Acc (2016) 135:187 DOI 10.1007/s00214-016-1937-2

2) M. C. E. Galbraith, S. Scheit, N. V. Golubev, G. Reitsma, N. Zhavoronkov, V. Despre, F. Lepine, A. I. Kuleff, M. J. J. Vrakking, O. Kornilov, H. Koppel and J. Mikosch, Nature Communications 8 1018 (2017)
